# Accuracy of Positive Airway Pressure Device—Measured Apneas and Hypopneas: Role in Treatment Followup

**DOI:** 10.1155/2013/314589

**Published:** 2013-08-25

**Authors:** Carl Stepnowsky, Tania Zamora, Robert Barker, Lin Liu, Kathleen Sarmiento

**Affiliations:** ^1^Health Services Research & Development Unit, Veterans Affairs San Diego Healthcare System, San Diego, CA 92161, USA; ^2^Department of Medicine, University of California, San Diego, CA 92037, USA; ^3^Pulmonary Service, Veterans Affairs San Diego Healthcare System, San Diego, CA 92161, USA; ^4^Department of Family and Preventive Medicine, University of California, San Diego, CA 92037, USA

## Abstract

Improved data transmission technologies have facilitated data collected from positive airway pressure (PAP) devices in the home environment. Although clinicians' treatment decisions increasingly rely on autoscoring of respiratory events by the PAP device, few studies have specifically examined the accuracy of autoscored respiratory events in the home environment in ongoing PAP use. “PAP efficacy” studies were conducted in which participants wore PAP simultaneously with an Embletta sleep system (Embla, Inc., Broomfield, CO), which was directly connected to the ResMed AutoSet S8 (ResMed, Inc., San Diego, CA) via a specialized cable. Mean PAP-scored Apnea-Hypopnea Index (AHI) was 14.2 ± 11.8 (median: 11.7; range: 3.9–46.3) and mean manual-scored AHI was 9.4 ± 10.2 (median: 7.7; range: 1.2–39.3). Ratios between the mean indices were calculated. PAP-scored HI was 2.0 times higher than the manual-scored HI. PAP-scored AHI was 1.5 times higher than the manual-scored AHI, and PAP-scored AI was 1.04 of manual-scored AI. In this sample, PAP-scored HI was on average double the manual-scored HI. Given the importance of PAP efficacy data in tracking treatment progress, it is important to recognize the possible bias of PAP algorithms in overreporting hypopneas. The most likely cause of this discrepancy is the use of desaturations in manual hypopnea scoring.

## 1. Introduction

 Obstructive sleep apnea (OSA) is a chronic medical condition requiring nightly application of therapy to effectively limit the number of apneas and hypopneas that would occur without intervention. The gold-standard treatment for OSA is continuous positive airway pressure therapy (PAP), which provides a pneumatic splint of the soft tissue in the upper airway [1]. PAP devices can measure and record airflow and pressure levels whenever the device is worn. They contain internal, proprietary (i.e., differing by manufacturer) algorithms that identify breathing disturbances and whether these disturbances are due to persistent obstructive or nonobstructive events. Thus, PAP devices can provide a measure of “residual” Apnea-Hypopnea Index (AHI) and its components, the Hypopnea Index (HI) and Apnea Index (AI). Although not equivalent to the indices measured by polysomnography or home sleep testing via Type III devices, the PAP terminology is nonetheless the same. 

American Academy of Sleep Medicine practice parameters and clinical guidelines recommend routine monitoring of adherence and efficacy data provided by PAP devices as an indication of treatment progress [2, 3]. Because residual AHI is primarily used to inform pressure changes and because its measurement by the PAP device is different relative to polysomnography (PSG) or Type III devices, it requires further study. PAP-scored AHI is different from that scored by PSD for two main reasons: (1) PAP measures are based solely on an airflow signal, and (2) they are based on an automated, proprietary algorithm. Several studies have examined PAP-scored AHI but have primarily attempted to evaluate the ability of the PAP device (autoadjusting PAP, in particular) to provide an initial baseline AHI value. Most have reported a strong correlation between PAP-scored AHI and manual-scored AHI [4, 5]. However, a certain percentage of AHI values would have resulted in different classifications, which can affect clinical management decisions.

A related but different issue concerns the accuracy of the Apnea-Hypopnea Index (AHI), as measured by the PAP unit in the home environment for the purposes of treatment efficacy (i.e., after a period of use). AHI accuracy is particularly important, given the increasing use of and reliance upon PAP data by providers, patients, and intermediaries (i.e., durable medical equipment staff). Ambulatory models of OSA care are gaining popularity, particularly the use of autotitrating PAP devices in lieu of in-laboratory CPAP titrations. In contrast to fixed pressure devices, which simply count the number of apneas occurring while PAP is applied, autoadjusting devices can make pressure changes based on the identification of these disturbances. With an ever-increasing demand for sleep apnea care, the ability to identify patients who may not be therapeutic on their PAP devices is critical. Efficacy of therapy is also an important factor in patient adherence. New technologies allow for data transmission directly from the PAP device to software accessible to the provider and, more recently, to the patients themselves (e.g., SleepMapper, Philips Respironics, Murrysville, PA). A variety of data transmission methods are possible, including the use of a smartcard, wired modem (via telephone line), wireless modem (via cellular network), and, more recently, Bluetooth modems to connect directly into home computers, tablets, or Smartphones. Remote monitoring is a trend within healthcare that is clearly accelerating, and in the sleep field, it facilitates the evaluation of compliance and efficacy of PAP therapy [6].

 Given the improved PAP data transmission technologies and resultant increased use of these data, we sought to investigate the accuracy of the PAP-measured AHI. We had the opportunity to conduct “PAP efficacy” studies in which participants wore PAP devices simultaneously with Type III cardiopulmonary recording equipment. Therefore, the goal of the present study was to specifically examine the accuracy of the identification of apneas and hypopneas by the PAP device.

## 2. Methods

### 2.1. Procedures

Twelve research participants from a larger trial evaluating a PAP adherence intervention were included in this study. The PAP adherence intervention study compared a usual care group to a group that was provided with extra education and clinical support via interactive website, phone calls, and in-person clinic visits [7]. They were also provided with daily access to their PAP data. Inclusion criteria for the study were purposefully broad and included those diagnosed with OSA (as defined by AHI >15 with predominately obstructive events) and prescribed PAP therapy. Participants who had a clinical indication for performing an efficacy study (e.g., either high residual PAP-measured AHI or subjective report that was inconsistent with PAP data) [3] were included. These participants underwent a home efficacy study, in which autoadjusting positive airway pressure therapy (APAP) devices was worn simultaneously with Embletta, a Type III cardiopulmonary recording device. 

### 2.2. Equipment Used

The Embletta (Embla, Inc., Broomfield, CO) was directly connected to the ResMed AutoSet S8 (ResMed, Inc., San Diego, CA) via a specialized cable that allowed for the direct recording of S8 data. Signals recorded include oximetry, chest effort, and body position. Airflow from the PAP device was used for scoring. RemLogic software was used for manual respiratory scoring. Apneas and hypopneas were manually scored according to the 2007 American Academy of Sleep Medicine guidelines, which included defining a hypopnea as being associated with a ≥4% oxygen desaturation [8]. AutoSet respiratory events were autoscored by the device, and summary statistics were obtained within RemLogic. Manual scoring was blind to the AutoSet-scored respiratory events. 

### 2.3. Data Analysis

Descriptive statistics (mean, median, and standard deviation and range) were calculated for the AHI, HI, and AI data. Scatterplots were generated to show the relationship between PAP-scored and manual-scored indices and included the line of identity. Spearman correlation coefficient was calculated. Wilcoxon signed rank test was used to test mean difference between the indices, and concordance correlation coefficients [9, 10] with 95% confidence interval (CI) were used to assess the agreement between PAP-scored and manual-scored indices. The concordance correlation coefficient less than 0.90 is interpreted as poor agreement, 0.90–0.95 as moderate, 0.95–0.99 as substantial, and greater than 0.99 as almost perfect [11]. Bland-Altman plots were created to provide a visualization of the bias and limits of agreement [12]. Data were analyzed using R [13].

## 3. Results

Participants were primarily overweight, middle aged, and sleepy with moderate to severe OSA (see Table 1) who had been using PAP on average 84.8 days prior to the PAP efficacy study. There were 6 men and 6 women. 

Mean PAP-scored AHI was 14.2 ± 11.8 (median: 11.7, range: 3.9–46.3) and mean manual-scored AHI was 9.4 ± 10.2 (median: 7.7, range: 1.2–39.3) (see Table 2). Paired sample means testing found differences between the PAP AHI and manual AHI (mean difference = 4.84, median difference = 4.25, *P* < 0.001) and between the PAP HI and manual HI (mean difference = 4.45, median difference = 3.5, *P* < 0.001), but not between PAP AI and manual AI (mean difference = 0.21, median difference = 0.4, *P* = 0.53). The correlation coefficient between PAP-scored and manual-scored AHI, AI, and HI was 0.93 (*P* < 0.001), 0.92 (*P* < 0.001), and 0.87 (*P* < 0.001), respectively. 

Concordance correlation coefficient is 0.87 (95% CI: 0.71–0.95) for AHI, 0.994 (95% CI: 0.98–0.998) for AI, and 0.50 (95% CI: 0.23–0.70) for HI. Based on published guidelines, the agreement between PAP and manual scoring is considered “poor” in AHI and HI and is “almost perfect” for AI. 

Ratios between the mean indices were calculated. The PAP-scored HI was 2.0 times higher than the manual-scored HI, the PAP-scored AHI was 1.5 times higher than the manual-scored AHI, and the PAP-scored AI was 1.04 of the manual-scored AI. It appears that the PAP device evaluated in this study, relative to manual scoring, only slightly overscored the number of apneas but significantly overscored the number of hypopneas. The difference in scoring of hypopneas seems to be the main contributor to the different AHI values between the PAP device and manual scoring. 

**Figure 1:**
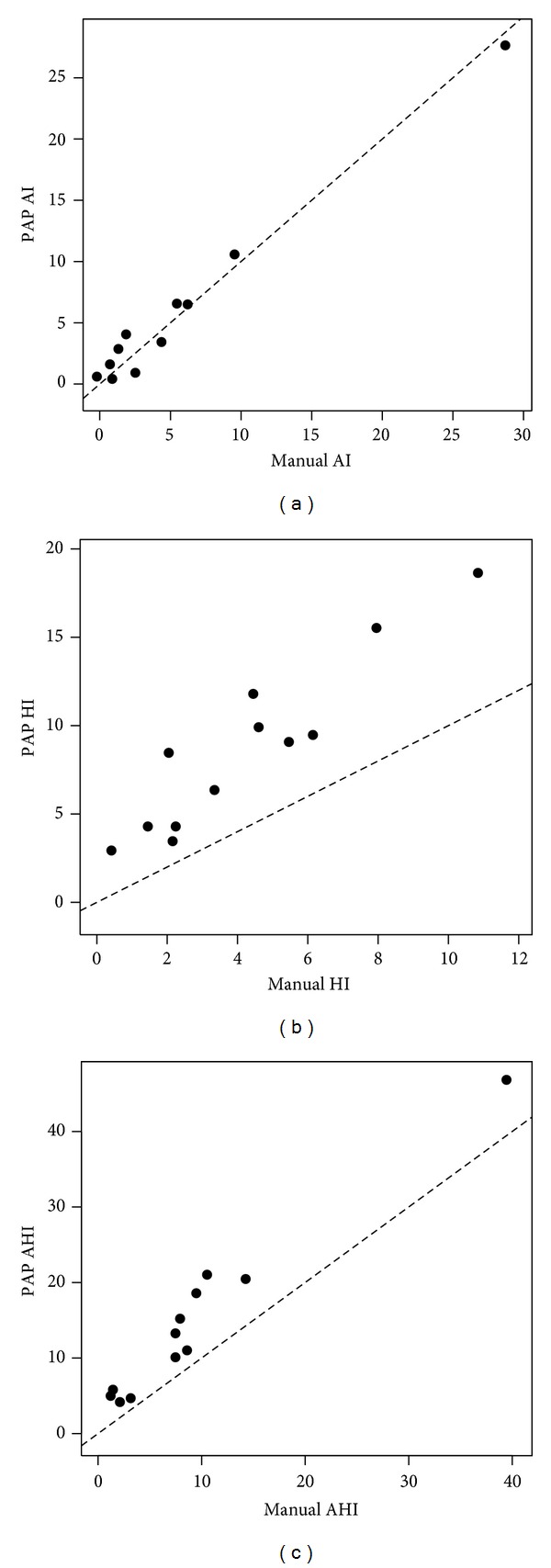
(a) Scatterplot of PAP-scored versus manual-scored Apnea Index. Each point represents one night. The diagonal line represents the line of identity. (b) Scatterplot of PAP-scored versus manual-scored Hypopnea Index. Each point represents one night. The diagonal line represents the line of identity. Note that all PAP HI values are greater than manual HI values. (c) Scatterplot of PAP-scored versus manual-scored Apnea-Hypopnea Index. Each point represents one night. The diagonal line represents the line of identity. Note that all values of PAP AHI are greater than manual AHI values.

**Figure 2:**
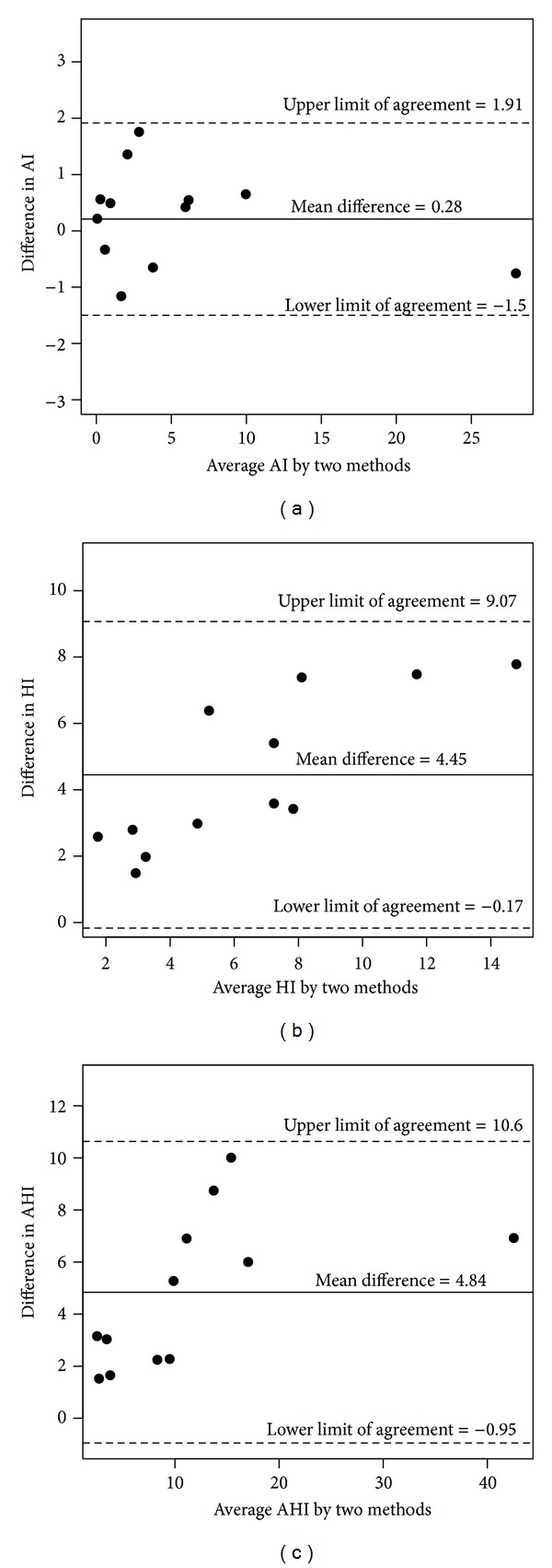
(a) Bland-Altman plot of PAP scoring versus manual scoring AI difference (PAP-scoring minus manual-scoring) by the mean. (b) Bland-Altman plot of PAP scoring versus manual-scoring HI difference (PAP scoring minus manual-scoring) by the mean. Note that all of the difference values were greater than 0, indicating that all PAP HI values were greater than the manual HI values. (c) Bland-Altman plot of PAP-scoring versus manual-scoring AHI difference (PAP scoring minus manual-scoring) by the mean. Note that all of the difference values were greater than 0, indicating that all PAP AHI values were greater than the manual AHI values.

Two graphical displays of the data were created. Figures 1(a)–1(c) show the scatterplots for the three indices, including the line of identity. In each case, the PAP-scored index was higher than the corresponding manual-scored index. Figures 2(a)–2(c) show the Bland-Altman plots. The mean bias (95% limit of agreement) is 4.84 (−0.95 to 10.6) for AHI, 0.28 (−1.50 to 1.91) for AI, and 4.45 (−0.17 to 9.07) for HI. 

## 4. Discussion

The practice of sleep medicine is evolving, and ambulatory models of sleep apnea management using home sleep testing and APAP therapy are not only noninferior to traditional evaluations but are also gaining wider acceptance by sleep providers [14]. Home sleep testing, or cardiorespiratory polygraphy, is indicated for the diagnosis of OSA in patients with a high pretest probability of moderate to severe OSA, to monitor efficacy of non-PAP therapies for OSA, and may be indicated in those who would otherwise not be recommended for home evaluation but who cannot undergo in-laboratory diagnostic testing [15]. Emphasis is placed on reviewing the raw data from home sleep tests to ensure accurate diagnoses. Similarly, review of downloaded data from PAP machines is of great importance in determining efficacy of therapy and should guide decisions to change PAP settings. Thus, in an era of increasing dependence on efficacy and compliance information in the clinical management of sleep apnea patients, a greater understanding of how to interpret this information is needed. 

In this study of home-based PAP efficacy, as measured by the S8 APAP device, the PAP-scored HI was on average more than double the manual-scored HI. Given the importance of PAP efficacy data in tracking treatment progress, it is important to recognize that this particular APAP device may overscore hypopneas. The most likely causes of this discrepancy are (a) the use of a proprietary algorithm and (b) the use of desaturations in manual hypopnea scoring. Because the number of apneas was underscored relative to manual scoring, the overall AHI does not appear to be different from manual scoring. This study and the evolving literature in this area suggest that it is important to understand how a specific PAP device identifies both apneas and hypopneas.

One previous study that used the S8 device also found relatively good apnea measurement but an overscoring of hypopneas [16]. That study found that the PAP HI was 3.3 times higher than the manual HI, and the resulting AHI was just over two times greater. Those values are slightly higher than the values found in the present study, but both speak to the importance of understanding the scoring algorithms for apneas and hypopneas of a specific PAP device so that treatment decisions are well informed. If it is found that, on average, a specific PAP device scores hypopneas at a rate of 2.0 times greater than manual scoring, then an adjustment can be made by the provider. For example, in the case where the measured HI is 20, the adjustment can be made by dividing 20 by the factor of 2 or an HI of 10 (which would theoretically be comparable to manual scoring).

Other studies in this area have utilized the RemStar autoadjusting PAP device by Philips Respironics. These study results show a different pattern, specifically that respiratory event detection varies based on the number of events. For example, RemStar-measured AHI tended to overestimate the AHI at lower AHI levels but underestimate the AHI at higher AHI levels [5, 17]. In short, it appears that AHI measurement is dependent on the specific APAP device used. 

If there are systematic differences between PAP devices, it is important for the field to request that the manufacturers provide clinicians and researchers with clear information regarding what level of adjustment is necessary to allow for the most accurate interpretation of the PAP-scored apneas and hypopneas. The PAP-scored AHI value is a useful data point for gathering information on therapeutic efficacy. Previous studies have examined the percentage of patients that continue to have residual OSA even while using a PAP device. In a study of patients using single-pressure CPAP, nearly 20% continued to have PAP AHI  >10 after 3 months [18], while in another study of patients undergoing a home APAP trial, 29% had PAP AHI  >10 [19]. The former study did not specify the CPAP device used, while the latter study used a ResMed AutoSet Spirit. Given the results of the current study and the associated literature, it would appear that the unique PAP device algorithms for automatic respiratory event detection affect the results of these and similar studies. Given the findings of the present study, it is possible that the study using the ResMed AutoSet has inflated AHI values, and therefore, the residual AHI in that study may be less than actually reported [19].

As per published clinical guidelines, the standard recommendation is that sleep monitoring is indicated for the assessment of treatment results on PAP therapy after (i) substantial weight loss (e.g., 10% of body weight) to ascertain whether PAP therapy is still needed at the prescribed pressure settings, (ii) substantial weight gain with return of symptoms (e.g., 10% of body weight) to ascertain whether pressure adjustments are needed, (iii) clinical response is insufficient (e.g., lack of symptom relief, above normal residual AHI, or poor adherence), or (iv) symptoms return despite a good initial response to CPAP [3, 20]. 

There are a number of potential study limitations. First, the number of participants is low relative to other studies in this area. That said, the results are consistent with the only other study that used the S8 APAP device. Second, the study was done in the home environment where there is less control than that in a laboratory environment. Third, there are limitations in scoring hypopneas with Type III home testing (events must meet flow and ≥4% saturation criteria but cannot be scored based on an arousal, as sleep is not measured). Finally, newer generation of PAP devices can distinguish central and obstructive events. Future studies can utilize this newer technology to see how well PAP algorithms can distinguish these events and what role they have in overall AHI values. 

 In summary, PAP devices have automated, proprietary algorithms for respiratory event detection. When event detection scoring is combined with PAP use duration in the denominator, a proxy AHI value is derived. Given the increased reliance on the PAP-scored events by both providers and patients, it is important to better understand the nuances of specific algorithms and how the PAP-scored AHI, HI, and AI values compare to those same values from manual scoring. Doing so is an important step toward making more informed treatment decisions. 

## Figures and Tables

**Table 1:** Baseline characteristics.

	Mean ± SD	Range
Age	62.0 ± 12.3	46–78
Body Mass Index (BMI)	30.4 ± 5.9	21.5–41.7
Apnea-Hypopnea Index (AHI)	42.9 ± 21.5	11.3–76.9
Epworth Sleepiness Scale (ESS)	14.2 ± 3.9	8.0–19.0

**Table 2:** OSA variables measured on efficacy study.

	Manual scoring		Autoscoring	
	Mean ± SD	Range	Mean ± SD	Range
Apnea-Hypopnea Index (AHI)	9.4 ± 10.2	1.2–39.3	14.2 ± 11.8	3.9–46.3
Apnea Index (AI)	5.1 ± 7.9	0–28.4	5.3 ± 7.7	0.20–27.6
Central Apnea Index (CAI)	2.6 ± 6.6	0–23.5	—	—
Obstructive Apnea Index (OAI)	1.5 ± 2.3	0–7.7	—	—
Mixed Apnea Index (MAI)	0.9 ± 1.3	0–4.6	—	—
Hypopnea Index (HI)	4.3 ± 3.0	0.5–10.9	8.8 ± 4.9	3.1–18.7
Oxygen Desaturation Index (ODI)	7.7 ± 7.4	0.6–27.2	—	—
